# Ecological momentary assessment of outcomes in allergic rhinitis and chronic rhinosinusitis: A review

**DOI:** 10.1002/alr.22982

**Published:** 2022-02-14

**Authors:** Rachel A. Schusteff, Margaret A. Chervinko, Sharmilee M. Nyenhuis, Victoria S. Lee

**Affiliations:** 1 Graduate Medical Education, College of Medicine, University of Illinois Chicago, Chicago, Illinois, USA; 2 Department of Ophthalmology and Visual Sciences, College of Medicine, University of Illinois Chicago, Chicago, Illinois, USA; 3 Department of Medicine, Division of Pulmonary, Critical Care, Sleep and Allergy, College of Medicine, University of Illinois Chicago, Chicago, Illinois, USA; 4 Department of Otolaryngology – Head and Neck Surgery, College of Medicine, University of Illinois Chicago, Chicago, Illinois, USA

**Keywords:** allergic rhinitis, chronic rhinosinusitis, ecological momentary assessment, symptoms

## Abstract

**Background:**

Allergic rhinitis (AR) and chronic rhinosinusitis (CRS) rely on patient-reported symptoms and quality-of-life measures, which are subject to bias. Ecological momentary assessment (EMA) captures data in real time through repeated short surveys, thus reducing errors/biases. EMA’s use in sinonasal conditions has not been well described, and the goal of this study was to examine the literature on EMA and AR/CRS.

**Methods:**

A literature review was performed using the following search terms: AR, CRS, and EMA. Inclusion criteria were the use of EMA reporting of sinonasal symptoms at more than one time point. Systematic reviews and non-full text articles were excluded. Population demographics, sinonasal disease, type of EMA platform used, type and severity of symptoms reported, medication use and symptom correlation with location/pollen/pollution were collected.

**Results:**

Eight studies met the inclusion criteria, and all focused on AR. All studies were conducted outside the United States in both children and adults. Seven studies used a smartphone application for reporting symptoms, and one used WeChat surveys. EMA data collection varied, with repetitive survey intervals determined either by patients (*n* = 6) or research team (*n* = 2). All studies reported sinonasal severity scores, while six reported additional symptoms (e.g., ocular, pulmonary, sleep, general health). Five collected self-reported allergy medication use. Seven studies correlated symptoms with location, pollen, or pollution.

**Conclusions:**

Few studies in AR and no studies in CRS assessed the use of EMA. EMA may provide a better understanding of the real-time relationship of environmental triggers with symptoms, in turn guiding treatment decisions.

## 1 | INTRODUCTION

Chronic rhinosinusitis (CRS) and allergic rhinitis (AR) are two of the most common sinonasal conditions. While the true prevalence is difficult to determine because clinicians rely mostly on self-report of symptoms, in the United States the prevalence of CRS is estimated at 2–14% and 11–33% for AR.^1,2,3^ In the United States, these two diseases combined account for at least $10.6 billion in direct costs per year and 64.5 million lost days from work.^1,2,3^

The diagnosis and management of CRS and AR primarily rely on self-reported symptoms at clinical visits when patients are typically asked to report their symptoms on average over a recent time period. Validated tools that are commonly used include the Allergic Rhinitis Control Test (ARCT) for AR and the 22-item Sinonasal Outcome Test-22 (SNOT-22) for CRS.^3,4^ Variability exists in the accuracy of self-reported symptoms as there is, most notably, a potential for recall bias. Further, this method of data collection does not capture more frequent variability that may occur based on moment-to-moment patient behaviors and environmental exposures. More broadly, AR symptoms are variable and can exhibit a seasonal pattern due to exposure to pollens/molds or a year-round pattern due to exposure to dust mites/pets/pests/indoor molds.^5^ Additionally, many people living with AR have a non-allergic component or mixed rhinitis, which is triggered by strong odors, pollutants, and/or smoke and is subject to frequent variability.^3^ Treatment of sinonasal conditions, such as specific types of rhinitis that have an environmental trigger component may rely on avoidance of triggers which can be variable and sometimes difficult to identify with traditional methods of data collection. Thus, tools are needed to capture sinonasal symptom variability and the environmental/personal exposures that occur at symptom onset.

Ecological momentary assessment (EMA) allows people to report their experiences/symptoms in real time, in real-world settings, over time, and across environmental settings.^6^ Utilizing technology such as written diaries, smartphone applications that collect real-time symptoms, and physiological sensors to gather information, EMA can greatly reduce recall bias and maximize ecological validity of clinical symptoms.^6^

EMA has been used in several chronic diseases to better understand the contextual factors associated with disease symptoms. For example, EMA has been used in asthma to assess symptoms and medication adherence and in depression to correlate negative/positive affect with stressors in everyday life.^7,8^ As sinonasal conditions heavily rely on patient-reported symptoms which are variable and are influenced by behaviors and exposures in the natural environment, EMA may be an applicable tool in the diagnosis and management of sinonasal conditions. Understanding the specific environmental triggers patients have can guide treatment options.^9,10^ The goal of this current study was the following: (1) to identify the studies on the use of EMA in CRS and AR, the two most common sinonasal conditions; (2) to describe study characteristics; and (3) to characterize outcomes measured and results.

## 2 | METHODS

### 2.1 | Search strategy

For this review, we conducted a comprehensive literature search on June 4, 2021. We searched three electronic databases (PubMed, Google Scholar, and ProQuest Dissertations & Theses A&I) (Table 1).

### 2.2 | Selection criteria

The study inclusion criteria were the following: (1) patients with ARor CRS, (2) utilization of EMA with follow up and reporting of sinonasal symptoms at more than one time point for at least some of those participating in the study, and (3) written in English or easily translatable. All types of EMA platforms, nonelectronic and electronic, were considered. Studies were excluded if they were: (1) systematic reviews, (2) those that did not have a full-text article, (3) protocol papers, and (4) abstracts. Systematic reviews were excluded as the goal of this study was to focus on original study data. In any case, the PubMed search only yielded three systematic reviews, which did not meet the inclusion criteria of being AR/CRS and utilizing EMA with reporting of sinonasal symptoms at more than one time point.

### 2.3 | Data extraction, analysis, and synthesis

M.A.C. (Professional Librarian/Research Specialist) performed the initial literature review and developed the search terms. R.A.S. (first author) initially screened the abstracts for inclusion. Of the included abstracts, fulltext articles were obtained and assessed for inclusion. All included and excluded full-text articles were reviewed by two reviewers to confirm eligibility (V.S.L. and S.M.N.). R.A.S. extracted the data from the included articles and compiled a table of outcomes and other variables found in each article. Population demographics, sinonasal disease, type of EMA platform used, type and severity of symptoms reported, medication use, and symptom correlation with location/pollen/pollution were abstracted from the full-text articles.

## | RESULTS

### 3.1 | Categories of study objectives

The data obtained from this review are split into three different categories and presented based on our study objectives: (1) search characteristics, (2) study characteristics, and (3) outcomes measured and results. Outcome categories are symptoms, medication use, environmental allergens and pollutants, feasibility of EMA, adherence to EMA, and symptom predictions.

### 3.2 | Search characteristics

**FIGURE 1:**
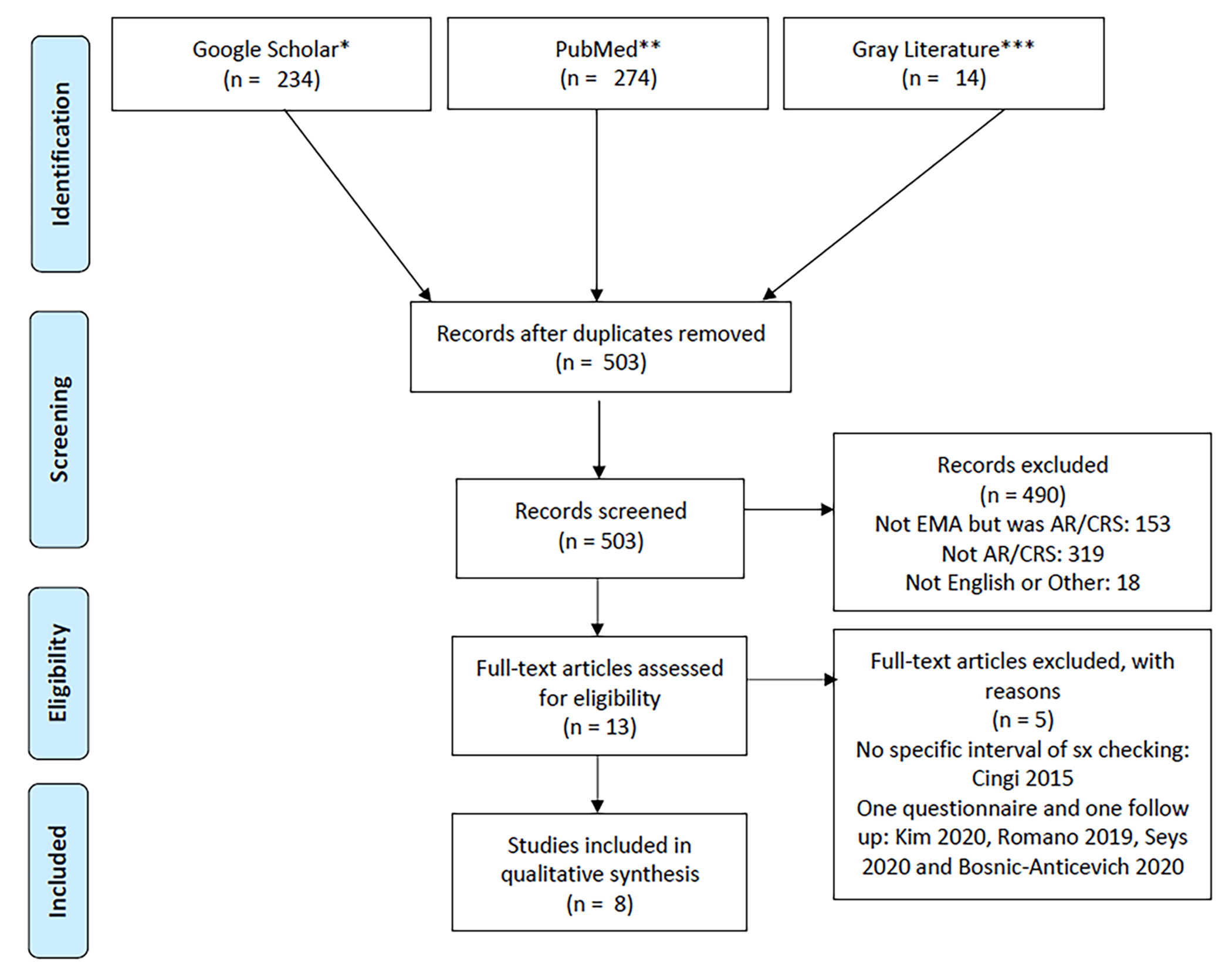
PRISMA Flow Diagram. * = Search: “smartphone” AND (rhinitis allergic OR seasonal “(questionnaire OR survey)”). ** = Search: ((((((((((allergic rhinitis[Other Term]) OR (“hay fever”[Other Term])) OR (“rhinitis, allergic”[MeSH Terms])) OR (“allergic rhinitis”[Title/Abstract])) OR (“hay fever”[Title/Abstract])) OR (“Rhinitis, Allergic”[Majr])) OR (“Rhinitis, Allergic, Seasonal”[Mesh] OR “Rhinitis, Allergic”[Mesh])) AND ((((((((“Smartphone”[Mesh]) OR “Cell Phone”[Mesh]) OR “Wearable Electronic Devices”[Mesh]) OR ((((“Self-Management”[Mesh]) OR “Self Report”[Mesh]) OR “Monitoring, Ambulatory”[Mesh]) OR “Ecological Momentary Assessment”[Mesh])) OR ((((((“self report”[Other Term]) OR (smartphone[Other Term])) OR (“self monitoring”[Other Term])) OR (“;ambulatory monitoring”[Other Term])) OR (“ecological monitoring”[Other Term])) OR (“self management”[Other Term]))) OR ((((“self monitoring”[Title/Abstract]) OR (“self report”[Title/Abstract])) OR (“ambulatory monitoring”[Title/Abstract])) OR (self reporting[Title/Abstract]))) ) OR (“ecological momentary assessment”[Title/Abstract]))) OR ((“Mobile Applications”[Mesh]) AND “Rhinitis, Allergic”[Mesh])) OR ((“app”[Title/Abstract]) AND (rhinitis[Title/Abstract]))) OR ((mobile[Title/Abstract]) AND (rhinitis[Title/Abstract] OR sinusitis[Title/Abstract])) Sort by: Publication Date. *** = Search: ab(Ecological momentary assessment) OR ab(self-regulation) OR ab(self monitoring) OR ab (monitor) AND ab(allergy OR allergies OR allergen OR allergic) OR ab(allerg*) OR ab(sinusitis OR rhinosinusitis

The searches yielded 503 results (*n* = 489 full-text articles, *n* = 14 grey literature; Figure 1). Of these, 490 were initially excluded because they were (1) not EMA related (*n* = 153), (2) not AR/CRS related (*n* = 319), or (3) not in English and could not be translated (*n* = 18). After this initial screen, 13 articles remained. Five were removed because they either sampled participants once (without follow-up) or did not document the interval for symptom checking (Figure 1). Eight articles were included in the final analysis.

### 3.3 | Study characteristics

#### 3.3.1 | Population demographics

(Table 2)

All studies were conducted outside the United States, specifically in Europe (*n* = 7)^11,12,14–18^ and in China (*n* = 1).^13^ Participants in the studies ranged from ages 0 to 92, with two studies^11,12^ not collecting or reporting the age of participants. Three of the eight studies solely focused on adults, while the other five had a mixed age population (pediatric and adult). All eight studies included patients with AR, and no studies included patients with CRS. AR diagnosis varied among studies, from, at the most basic level, patient self-report^12,15,16,17,18^ to the criterion of positive allergy testing results.^11,13,14^

#### 3.3.2 | EMA platform type (Table 2)

All studies used an electronic EMA platform. Xi et al.^13^ was driven by clinician prompting of participants to complete symptom scores at the same time daily, using WeChat surveys to collect data. Six other studies^11,12,15–18^ relied on participants to self-manage symptom reporting, using smartphone applications to collect data. Di Fraia et al.^14^ prompted participants to input symptom data when pollen levels were high, also using a smartphone application to collect data. If study participants missed two or more consecutive days of reporting, they would get an automatic alert message and if they missed more than four consecutive days, they received a phone call.^14^

### 3.4 | Study outcomes and results (Table 2)

#### 3.4.1 | Symptoms

All studies used EMA to report sinonasal symptoms and their respective severities. EMA was also used to report ocular and pulmonary symptom severity scores in six studies.^11,12,14–17^ In addition to sinonasal/allergy symptoms, several studies reported nonatopic symptoms such as overall wellness or wellbeing^12^ and work productivity.^15^ One study^13^ reported symptoms of insomnia, mood, and work behaviors.

#### 3.4.2 | Medication use

Six studies reported allergy medication use using EMA. All six studies asked participants if they took allergy medications,^11,12,14,16,17,18^ and one also used antihistamine sales data.^16^ Self-reported medication use was correlated with increased grass pollen^11^ and increased symptom severity.^12^ Medication use assessed via antihistamine sales were correlated with both symptom severity and pollen levels.^16^

#### 3.4.3 | Environmental allergens and pollutants

Six studies focused on outdoor allergens.^11,13,14,16–18^ Five studies looked at symptoms related to outdoor allergens only,^11,13,14,16,18^ with one of these focusing on fall season outdoor allergens^13^. Bédard et al.^17^ studied both indoor and outdoor allergens.

Five studies^11,13,14,16,18^ reported pollen levels specifically, while Bédard et al.^17^ also reported particulate matter and ozone pollutant levels. Geolocation was used to determine these levels in three studies.^11,14,17^ In two studies,^12,15^ geolocation data was collected but not used to determine these levels. In the three studies that obtained these levels but did not collect geolocation data,^13,16,18^ levels were collected at general locations where participants in the study lived. In the six studies^11,13,14,16–18^ that collected data on pollen/pollutant levels, all found a positive correlation between pollen/pollutant levels and worse sinonasal symptom severity.

#### 3.4.4 | Feasibility of EMA

Two studies looked at the feasibility of using EMA to report sinonasal symptoms in those with AR.^12,16^ Vigo et al.^12^ determined that EMA was feasible to collect data about sinonasal symptoms because they found a strong correlation with the number of antihistamines prescribed and the reported well-being of the participants in the study.^12^ De Weger et al.^16^ concluded that it was feasible to use EMA to monitor changes in symptom severity in those with AR because they found that symptom scores were correlated with pollen levels and the sale of prescription antihistamines in The Netherlands.^16^

#### 3.4.5 | Adherence to EMA

Di Fraia et al.^14^ measured the adherence to EMA symptom reporting by participants for the duration of the study. Adherence was the highest in the first 6 days of the study and was higher during “peak pollen seasons.”

#### 3.4.6 | Symptom predictions

Silver et al. was the only study included in our review that used “skill models” to determine if the use of EMA could predict next-day sinonasal symptoms in those with AR.^18^ They showed that those with milder sinonasal symptoms were located farther from pollen sites.

## 4 | DISCUSSION

Although EMA is an emerging tool to assess symptoms in real time, our literature search found very few studies that utilized EMA to assess symptoms in AR and no studies using EMA in CRS. The existing studies, however, do show that in those with AR it is feasible to use EMA to report sinonasal symptoms in real time.

Currently, the diagnosis of CRS and AR are based on clinical history and/or validated tools such as the SNOT-22 and ARCT.^3,4^ Both the patient history and the validated tools rely on patients to remember previous symptoms and potential allergen triggers, which can result in recall bias. Utilization of EMA for AR/CRS may potentially be useful to aid in the diagnosis of these conditions because it would allow clinicians to assess symptoms and correlate symptoms with environmental triggers in real time. Additionally, our review demonstrates that it is feasible to use EMA to report sinonasal symptoms in real time on a large scale. It would, however, be beneficial to study the feasibility of using EMA in a small patient population with unknown allergen triggers to determine if reporting sinonasal symptoms in real time can elucidate specific allergen triggers for these patients.

The few existing studies looking at the use of EMA to evaluate AR were quite heterogeneous. The outcomes measured and results presented included symptoms,^11–18^ medication use,^11,12,14,16–18^ environmental allergens and pollutants,^11,13,14,16–18^ feasibility of EMA,^12,16^ adherence to EMA,^14^ and symptom predictions.^18^ In addition, we found heterogeneity in the age of participants and location of the studies. Environmental triggers vary widely by geographic location, and our review revealed no studies conducted in the United States. Additionally, the majority of studies correlated EMA-reported symptoms with outdoor environmental exposures (e.g., pollen, pollutants), highlighting a need for studies that further explore the impact of indoor environment specifically on sinonasal conditions.

The type of EMA platform used also varied widely among studies. Seven studies^11,12,14,15,16,17,18^ used smartphone applications. Two studies^15,17^ used the same smartphone application, whereas five studies^11,12,14,16,18^ used different smartphone applications. Xi et al.^13^ used WeChat to communicate with study participants. Given the use of several different smartphone applications for reporting symptoms, we found heterogeneity among the interfaces and symptoms reported in these smartphone applications. Future studies using EMA in sinonasal disease should consider utilizing standardized symptom questions so comparisons can be made across studies.

EMA has been used to successfully aid in the diagnosis and treatment of neurological and psychiatric conditions.^19–25^ For example, in bulimia, EMA was used to predict binge eating behaviors, helping clinicians identify when patients are restricting calories so they can predict when binges will occur.^19^ In sinonasal disease, however, this review demonstrates that the existing literature is limited and quite heterogeneous in many aspects, including aims, type of EMA used, and type of data collected. There are, however, multiple potential intriguing applications for EMA in the diagnosis and management of sinonasal conditions. In theory, EMA data can be used to correlate symptoms and pollen counts, not only to aid in diagnosis but also to evaluate the clinical relevance of a patient’s sensitization to a specific pollen. EMA datasets also contain a wealth of real-time information compared with standard methods of assessment, and these datasets are robust enough to generate short-term symptom prediction models that can help develop tailored timing of medication regimens. There is also data to suggest the act of EMA itself improves medication adherence.^9,10^

Our study was limited, as it was not conducted as a systematic review. One reason we chose to conduct a narrative review instead is that we were limited by study heterogeneity as detailed above. Our study was also limited by individual participant adherence to the interventions. In addition, if participants became aware of pollen/pollutant counts before reporting their daily symptoms, this could have created bias in patient-reported symptoms. No studies met our criteria in CRS, and more research is needed here.

## 5 | CONCLUSIONS

We demonstrated in this review through the use of eight different studies how EMA can be used in reporting sinonasal symptoms in AR. Type of EMA platform used, medications reported, aims of the different studies, and additional allergic symptoms reported all contributed to the heterogeneity in these studies. Our study also highlights the lack of EMA use in CRS. These findings, along with further research in AR and CRS using EMA can help to determine specific allergen triggers and aid in the diagnosis of these conditions.

## Figures and Tables

**TABLE 1:** Search terms used in different databases searched

Database	Search terms
PubMed	*((((((((((allergic rhinitis[Other Term]) OR (“hay fever”[Other Term])) OR (“rhinitis, allergic”[MeSH Terms])) OR (“allergic rhinitis”[Title/Abstract])) OR (“hay fever”[Title/Abstract])) OR (“Rhinitis, Allergic”[Majr])) OR (“Rhinitis, Allergic, Seasonal”[Mesh] OR “Rhinitis, Allergic”[Mesh])) AND ((((((((“Smartphone”[Mesh]) OR “Cell Phone”[Mesh]) OR “Wearable Electronic Devices”[Mesh]) OR ((((“Self-Management”[Mesh]) OR “Self Report”[Mesh]) OR “Monitoring, Ambulatory”[Mesh]) OR “Ecological Momentary Assessment”[Mesh])) OR ((((((“self report”[Other Term]) OR (smartphone[Other Term])) OR (“self monitoring”[Other Term])) OR (“ambulatory monitoring”[Other Term])) OR (“ecological monitoring”[Other Term])) OR (“self management”[Other Term]))) OR ((((“self monitoring”[Title/Abstract]) OR (“self report”[Title/Abstract])) OR (“ambulatory monitoring”[Title/Abstract])) OR (self reporting[Title/Abstract])))) OR (“ecological momentary assessment”[Title/Abstract]))) OR ((“Mobile Applications”[Mesh]) AND “Rhinitis, Allergic”[Mesh])) OR ((“app”[Title/Abstract]) AND (rhinitis[Title/Abstract]))) OR ((mobile[Title/Abstract]) AND (rhinitis[Title/Abstract*] OR sinusitis[Title/Abstract])) Sort by: Publication Date
Google Scholar	*“smartphone” AND (rhinitis allergic OR seasonal “(questionnaire OR survey)”)*
ProQuest Dissertations & Theses A&I	ab(Ecological momentary assessment) OR ab(self-regulation) OR ab(self monitoring) OR ab (monitor) AND ab(allergy OR allergies OR allergen OR allergic) OR ab(allerg*) OR ab(sinusitis OR rhinosinusitis)

**TABLE 2:** Summary of search results

Authors	Study population	Intervention	Comparator	Outcome(s)	Timing	Results
Bastl et al.^11^	Users of the Patient Hayfever Diary (PHD) app with a pollen allergy in Germany (*n* = 1823) and Austria (*n* = 1465) with significant positive correlation to birch tree or grass pollen locations and who completed at least 15 data entries	Users rated severity of eye, nose, and lung symptoms, severities from 0 to 3 for a total max. score of 9 (most severe) and entered medications in the PHD app	None	- PHD score - Symptom load index of PHD score - EMA symptoms - Symptom load index of EMA	10 years	- Strong correlation with symptom scores and pollen levels (r > 0.9) - High correlation with the nasal symptoms - Medication use was higher during grass pollen season
Vigo et al.^12^	425 participants with self-identified allergy symptoms who downloaded the Britain Breathing App	Participants rated daily symptoms (nasal, eyes, breathing), overall wellness, and medications. Every report had a time stamp and a location from phone’s GPS.	None	- Allergy symptoms (nasal, breathing, eye) - Overall wellness symptoms - Current allergy medications - Antihistamines prescribed	8 months	Nasal symptoms were correlated with overall wellness (r = 0.75), eye symptoms (r = 0.62) and breathing symptoms (r= 0.57; *p* < 0.0001) - Medication use associated with lower overall wellness 0.73 vs. 0.47 (*p* < 0.0001) - Positive correlation with number of reports per week and average lack of wellness (r = 0.73, *p* < 0.05)
Xi et al.^13^	Adults with seasonal AR with a regular work schedule, positive specific IgE to fall-prevalent pollens only	Measured pollen at 13 sites in Beijing daily for 2 weeks. Participants completed questionnaires regarding nasal symptoms, sleep and insomnia habits, moods, and unethical work behaviors through WeChat daily	None	- Average daily pollen concentration for the 2 weeks - Nasal symptom score - Jenkins Insomnia Scale - Mood - Willingness of patients to commit unethical behaviors	2 weeks	- Men had more severe rhinitis symptoms and negative moods than women - Younger patients had more severe rhinitis, insomnia, and negative mood symptoms - Pollen concentrations positively impacted rhinitis symptoms (*p* < 0.001) - AR symptoms positively impacted insomnia symptoms (*p* < 0.01) and negative mood (*p* < 0.05) - Pollen levels had an effect on all symptoms of unethical work behaviors, insomnia, AR symptoms, and mood (*p* = 0.07)
Di Fraia et al.^14^	101 chil- dren/adolescents (age 10–18 years) in Rome, Italy and 93 adults (> 18 years) in Pordenone, Italy Being followed up for at least one year for allergic rhinoconjunctivitis (objectively confirmed by skin prick tests or in vitro immunoglobulin E tests, or both) due to outdoor aeroallergens, living within 30 km of aerobiological study center	T0 (first clinical assessment): nasal, lung, and eye allergy symptoms and daily medication intake collected using mobile app (Allergy Monitor), which was based on their locations and suspected high pollen counts T1 (second clinical assessment): repeated questionnaires and app diaries	None	- Rhinoconjunctivitis Total Symptom Score - Combined Symptom and Medication Score VAS score - Diary adherence	90 days	- Adherence was highest during the first 6 days of the “prescribed period” (93.1%) and declined to 83.7% at roughly 40 days and then 78.6% at roughly 75 days - Adherence could be predicted during the first 3 weeks (*p* < 0.001) for both age groups - Adherence was higher during peak pollen seasons
Bousquet et al.^15^	4129 Allergy Diary app users with AR symptoms, ages 12–92 years old from 19 countries	Users input their symptoms and were evaluated in five global symptom categories using a VAS (allergic symptoms, nasal symptoms, eye symptoms, asthma symptoms, and work productivity)	None	- VAS global symptom scores - Individual symptoms	1 year	- AS scores were strongly correlated with nasal symptoms (r > 0.88), eye symptoms (r > 0.71) and asthma (r > 0.55)
de Weger et al.^16^	Approximately 500 adults in the Netherlands who used the Allergieradar.nl app and had symptoms of AR	The Allergieradar.nl app collected data on allergy symptoms (nose, eye, and lung), medications used, previous allergy testing to grass or tree pollen. Daily pollen counts were collected in Leiden, Netherlands	None	- Daily pollen counts - Severity of allergy symptoms (nose, eye, lung) - Allergy medication type - Sales of antihistamines in the Netherlands from 2009 to 2012	3 years	- Daily nose and eye symptoms were > lung symptoms - Highest peak in symptom scores was in the birch tree pollen season - 85% of the participants used oral medication and/or nasal sprays (67%) - Antihistamine prescription sales correlated with the average symptom scores (r = 0.88)
Bédard et al.^17^	3323 European MASK app users with self-reported AR who authorized geolocation	Patients downloaded the Allergy Diary App, recorded symptoms of AR and allergies (global symptoms, nasal symptoms, eye symptoms and asthma) daily, medications daily	Pollen versus - no-pollen season -	- Allergic symptoms, analyzed as VAS score - Need for medications - Pollutant (ozone and particulate matter)levels - Pollen levels	20 months	- Allergic symptoms are worse when ozone levels and grass pollen specifically were high (*p*< 0.001)
Silver et al.^18^	Users of pollen forecast apps with AR symptoms in Melbourne and Canberra, Australia that were within 50 km (Melbourne) and 20 km (Canberra) of the pollen collection sites	Daily pollen levels were measured in Melbourne and Canberra. These were forecast to an app where its users could report rhinitis symptoms and optionally report asthma symptoms and medication use	None	- AR daily symptom severity (1–5) reported as a mean symptom score (MSS) - Pollen count predictions - Actual pollen counts (low, medium, and high)	26 months	- Mean daily rhinitis symptom scores were positively correlated with the pollen concentrations in Melbourne (0.45) and Canberra (0.50) - Symptom scores increased with increasing pollen concentrations - Symptoms were worse in the morning between 5 and 10 a.m. and between from 3 and 8 p.m. - Milder symptoms were found farther from the pollen collection sites

Abbreviations: AR, allergic rhinitis; EMA, Ecological Momentary Assessment; VAS, visual analogue scale.
